# Mechanical response of a surface of increasing hardness covered with a nonuniform polymer brush: a numerical simulation model†

**DOI:** 10.1039/d0ra01385d

**Published:** 2020-04-01

**Authors:** J. S. Hernández-Fragoso, S. J. Alas, A. Gama Goicochea

**Affiliations:** Posgrado en Ciencias Naturales e Ingeniería, Universidad Autónoma Metropolitana Unidad Cuajimalpa Ciudad de México 05300 Mexico salas@correo.cua.uam.mx; Departamento de Ciencias Naturales, Universidad Autónoma Metropolitana Unidad Cuajimalpa Ciudad de México 05300 Mexico; Departamento de Química, Universidad Autónoma Metropolitana Unidad Iztapalapa Av. San Rafael Atlixco 186 Ciudad de México 09340 Mexico; División de Ingeniería Química y Bioquímica, Tecnológico de Estudios Superiores de Ecatepec Av. Tecnológico s/n Ecatepec Estado de México 55210 Mexico agama@alumni.stanford.edu

## Abstract

The compression force with indentation on a polymer brush with chains of unequal lengths is predicted with numerical simulations, as a function of increasing hardness of the grafting surface, finding that properties of the brush are distinguished from those of the surface and that its hardness propagates through the brush.

## Abstract 2

The contributions to the force on a deformable surface covered with polymer brushes can be accounted for separately.

## Graphical abstract

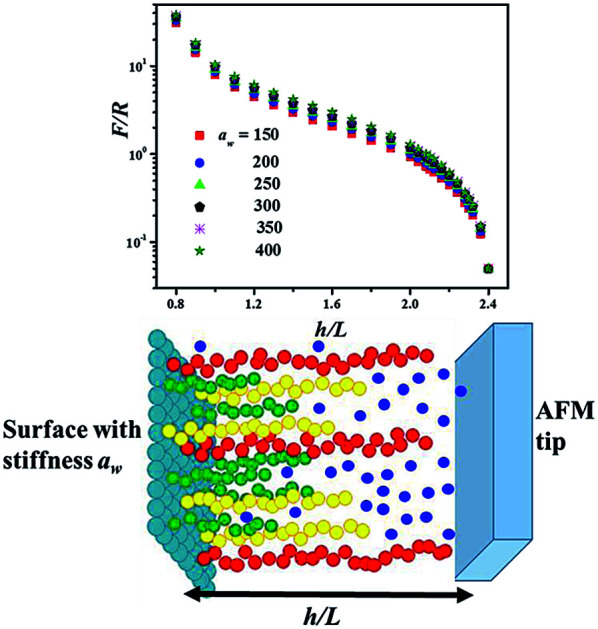

Polymer brushes^1^ are useful for many applications, such as to control interactions between nanoparticles^2^ and to build functional interfaces for smart coatings;^3^ they are present in cell membranes and bone cartilages,^4^ as slip agents for plastic sheets^5^ and in drug delivery.^6^ Although most studies have been carried out for uniform polymer brushes, *i.e.*, those made up of chains of the same length, some have been performed for brushes made up of polydisperse chains.^7–9^ Nonuniform brushes are used as coatings for stimuli – responsive nanoparticles^10^ and in the design of stable colloidal dispersions.^11^ From the basic research point of view, they have also attracted attention because scaling laws of properties such as brush thickness and grafting density differ from those of uniform brushes.^12,13^ In addition to these works, atomic force microscopy (AFM) studies first carried out by Sokolov's group^14–16^ showed that the surface of cancerous human cervical epithelial cells are covered by chain – like structures of different length that can be described as a nonuniform “polymer” brush. The compression force on these polydisperse brushes was found to be lower than that on comparable monodispersed brushes on healthy cervical epithelial cells.^14,17^

Extracting specific information from brushes, such as grafting density, chains' polymerization degree and brush thickness is difficult experimentally because the measuring techniques, such as X-ray reflectivity and even AFM, can measure only averaged values.^18^ Numerical modelling^19^ is a useful tool that can help identify these brush characteristics and predict phenomena not easily accessible in experiments, such as the brush structural conformation under compression and the force dependence on compression degree. Atomistically detailed simulations are very accurate but to model nonuniform brushes under varying compression degree they require large systems with long computational time. An alternative approach is to use mesoscopic scale simulations, where some of the atomic – level degrees of freedom are coarse – grained, thereby reducing the time required to carry out the calculations. One of the most popular mesoscale techniques used to predict properties of soft matter systems is dissipative particle dynamics (DPD).^20,21^ In DPD atoms or molecules are grouped into beads whose interactions are modelled by simple, repulsive and short-range laws yielding accurate results for relatively small systems in short simulation time. DPD has been successfully used to model uniform and nonuniform polymer brushes^22–25^ but so far only the properties of the brushes have been studied. There are no reports, to the best of our knowledge, on the effect of varying the stiffness of the surface on which the chains are tethered. The purpose of this paper is to test *via* DPD simulations whether increasing the stiffness of a surface with grafted chains modifies the force on the brush while keeping the properties of the former unchanged. Our work is motivated by the need to interpret AFM experiments on the force experienced by human cancerous cervical cells covered by polymer – like brushes of nonuniform length.^14–16^ It is known that the stadium of the disease in cells like these correlates with a transformation of their surface, resulting in stiffer cellular membranes.^26^ Thus, it is necessary to account for the separate contribution of the surface and the brush in force profiles.

Fig. 1 shows a schematic representation of our simulations' setup. Polymer chains of three lengths (in green, yellow and red in Fig. 1) are built following the Kremer–Grest bead-spring model,^27^ and grafted on a surface (in blue in Fig. 1) defined by an effective force law *F*_w_(*z*) = *a*_w_(1 − *z*/*z*_C_).^28^ The stiffness of the surface is increased by increasing the value of the parameter *a*_w_, where *z* is the component along the *z*-axis of the position of a fluid particle (brush bead, solvent bead), perpendicular to the plane of the surface and *z*_C_ is a cut-off distance beyond which *F*_w_(*z*) = 0. To model the tip of the AFM, represented by the hemisphere in Fig. 1, we used also the short-range surface force law, with *a*_w_ fixed in all cases. Typically, AFM probes are micron sized^14^ while the lateral side of the samples are of order nm, hence the curved tip can be approximated by a flat surface at short distances. This setup has been used to predict successfully the force-indentation profiles on nonuniform brushes.^17^

**Fig. 1:**
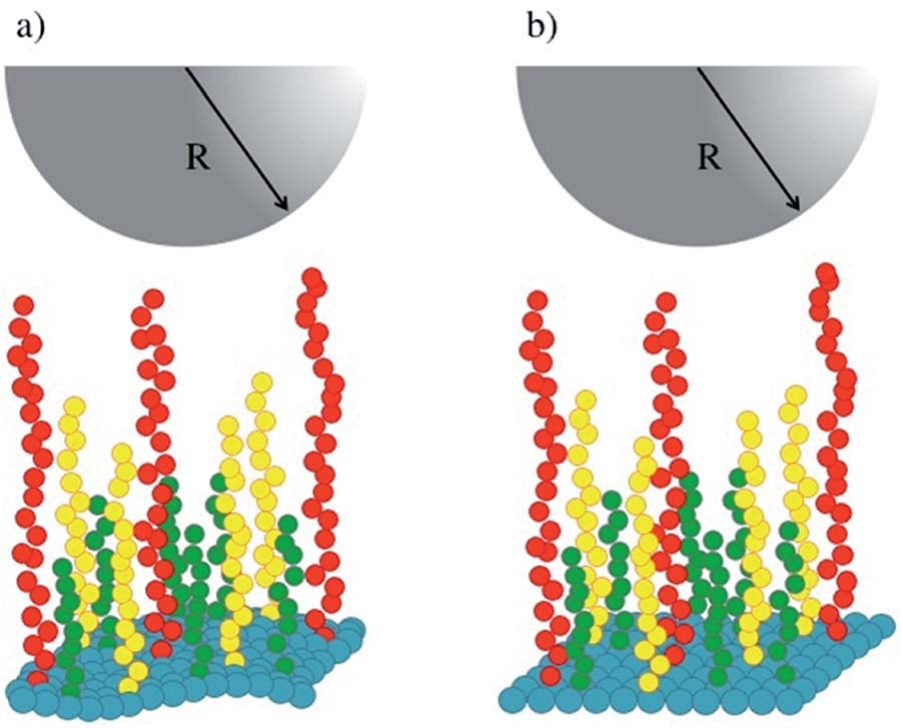
Schematic illustration of the system studied in this work. (a) A soft surface (blue beads) is covered with chains of three lengths, represented by green, yellow and red beads. (b) The stiffness of the surface is increased while keeping the rest of the system identically as in (a). The tip of an AFM is represented by the grey hemisphere. The solvent fills the rest of the simulation box and is not shown for simplicity. See the ESI for full details.†

Once the system shown in Fig. 1 is built, the simulations proceed as follows. Starting from a maximum separation between both surfaces, where the brushes are unperturbed by the AFM tip (upper surface in Fig. 1) for a given value of the chains grafting surface hardness (*a*_w_), DPD simulations are run at constant chemical potential, volume and temperature. After the equilibrium phase is finished, the component of the pressure tensor along the compression direction (*P*_*zz*_, along the *z*-axis) is calculated and averaged during the production phase, and is recorded for that value of the separation between the surfaces. Then, the distance between the surfaces is reduced by a constant amount while keeping the rest of the system the same and new simulations are run to obtain the value of *P*_*zz*_ at the new distance. The procedure is repeated for increasing compression (reduced surface-to-surface distance) until the maximum compression while maintaining equilibrium is reached. This procedure yields a force *vs.* compression curve for a particular value of the surface stiffness *a*_w_. To obtain a curve for a stiffer surface we increase *a*_w_ and new simulations are performed at the same series of distances, for comparison. It must be emphasized that only the surface-to-surface distance and the grafting surface stiffness are changed; everything else is kept constant, including the properties of the brushes. As the compression is increased the number of solvent beads is reduced so that the chemical potential remains constant. Full details about the model, the simulation protocol and other details can be found in the ESI.†

The structure of the fluid made up of brush and solvent beads is presented in Fig. 2, which shows the bead concentration profiles for a particular distance between the surfaces, *h*, as the grafting surface stiffness parameter *a*_w_ is increased. The brushes in Fig. 2 are only slightly compressed, as signaled by the small maxima in their profiles in the region 9 ≤ *h*/*r*_c_ ≤ 12, where *r*_c_ is the DPD cut-off length. In Fig. 2 the brushes grafting surface is on the left (*h*/*r*_c_ = 0) and the flat tip of the AFM is on the right (*h*/*r*_c_ = 12). There are plenty of solvent beads near the AFM tip because the compression is low. As the compression increases the number of solvent beads is reduced and for the maximum compression the solvent's concentration profile is below the brushes' concentration profile, see also the ESI.† Notice the brushes' profiles are approximately parabolic, in agreement with scaling theories^29,30^ and with numerical simulations using models different from DPD.^31^

**Fig. 2:**
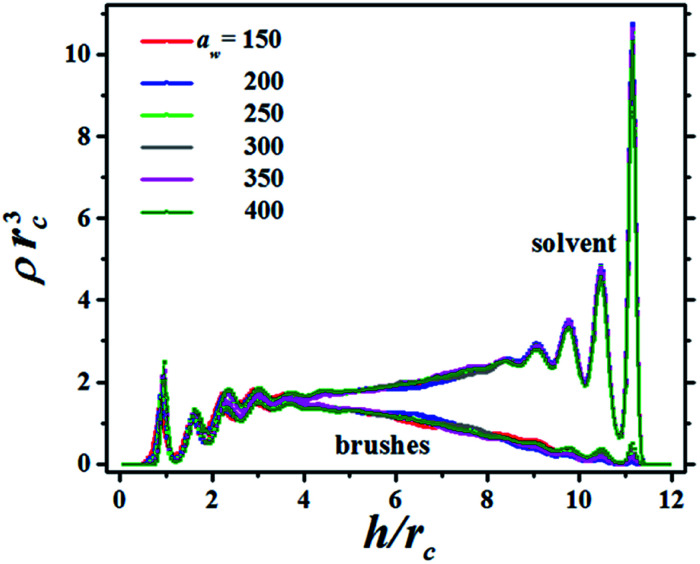
Concentration profiles (*ρ*) of the brushes and solvent for increasing values of the stiffness of the grafting surface, *a*_w_, which is reported in dimensionless units. The distance between the grafting surface (on the left, at *h*/*r*_c_ = 0) and the flat tip of the AFM (at *h*/*r*_c_ = 12) is represented by *h* and is normalized by the DPD cut-off distance, *r*_c_, to render it dimensionless.

To compare with experiments using AFM where the force on the brush (*F*) is usually normalized by the radius of the probe (*R*), we use the so called Derjaguin approximation:^32^
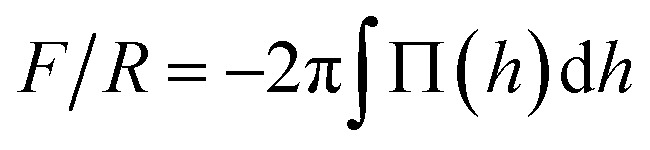
, where Π(*h*) = *P*_*zz*_(*h*) − *P*_B_ with *P*_B_ being the bulk pressure of the uncompressed brush and the integral is evaluated between the maximum and minimum compression distances. This approximation considers two spheres whose radii are much larger than the distance separating the surfaces of those spheres. Then the net force between them is obtained from the sum of the forces between thin, circular flat strips on the surface of the spheres. The resulting force is proportional by a geometric factor to the energy (per unit area) required to keep the surfaces a given distance apart.^32^ Derjaguin's approximation works well for short range interactions and it has been verified experimentally in numerous occasions. The Derjaguin approximation is useful to express the force on finite surfaces with curvature in terms of the force between semi-infinite plane surfaces.^32^

In Fig. 3 the force profiles obtained for all values of the stiffness of the brushes grafting surface are shown, which are obtained after extensive simulations at each compression distance. To improve the comparison with experimental trends we have normalized the distance between surfaces (*h*) by the average thickness of the brushes under no compression, *L*, which is defined by 
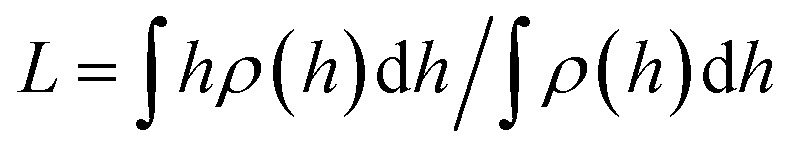
.^33^ The profiles of the three chain sizes are included in *ρ*(*h*). Two compression regimes are clearly identified in Fig. 3, which are indicated by the lines. The origin of these two compression regimes comes from having a relatively dense brush (grafting density equal to 1.76 nm^−2^), made up of the shortest chains with 5 beads, along with two brushes of larger polymerization degree. These short chains give rise to the strong compression regime. The larger chains, with polymerization degrees equal to 30 and 42 beads, and grafting densities equal to 0.49 nm^−2^ and 0.20 nm^−2^, respectively, are less dissimilar between them and constitute the second compression regime. The stiffness of the surface is reported in reduced units. Once it is multiplied by (*k*_B_*T*/*r*_c_) the surface stiffness varies in the range 0.96 nN ≤ *a*_w_ ≤ 2.56 nN. In addition to the two compression regimes in Fig. 3 there is also a growing force within each of them as the surface stiffness is increased. Notice that the strongest compression regime ends where the distance between surfaces equals the average brush thickness, *i.e.* when *h* = *L*, while the weaker compression range goes from 1 ≤ *h*/*L* ≤ 2. Thus, the nonuniform brush behaves under compression as if it were made up of two effective brush lengths. To analyse the results from Fig. 3 we have expanded the force profiles in the two compression regimes separately in Fig. 4, focusing only on the profiles for the softest (*a*_w_ = 150) and stiffest (*a*_w_ = 400) surfaces, for simplicity.

**Fig. 3:**
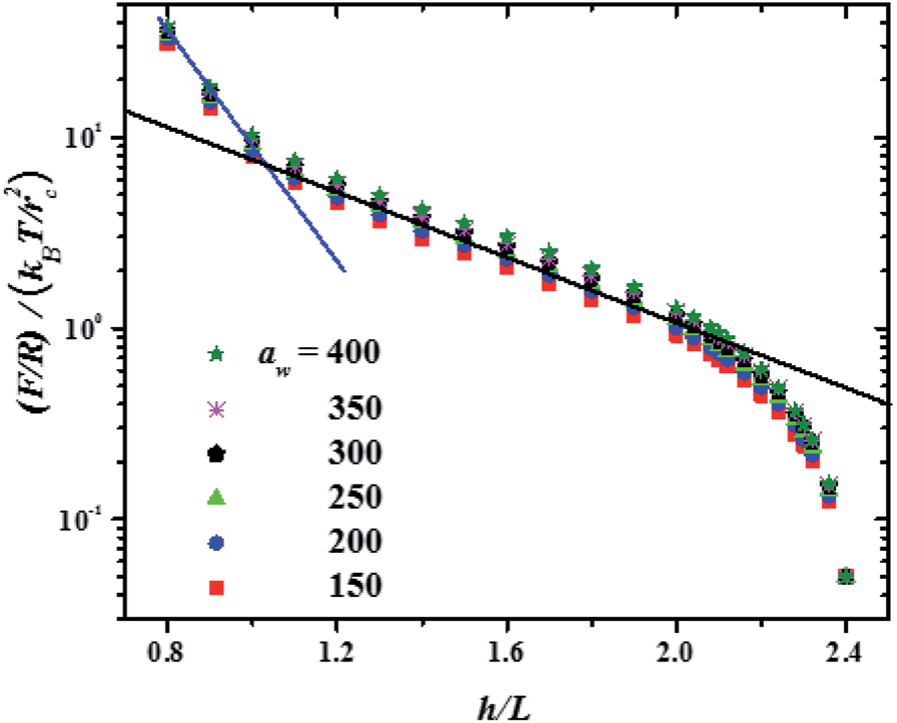
Force profiles for increasing grafting surface stiffness (*a*_w_, in reduced DPD units). *R* is the radius of the AFM probe and *L* is the averaged thickness of the unperturbed brushes. See text for details. To render *F*/*R* dimensionless the *y*-axis is divided by the thermal energy over *r*_c_^2^. *T* is the absolute temperature and *k*_B_ is Boltzmann's constant. Error bars are smaller than the symbols' size. The black and blue lines are meant only to indicate that there are two compression regimes.

**Fig. 4:**
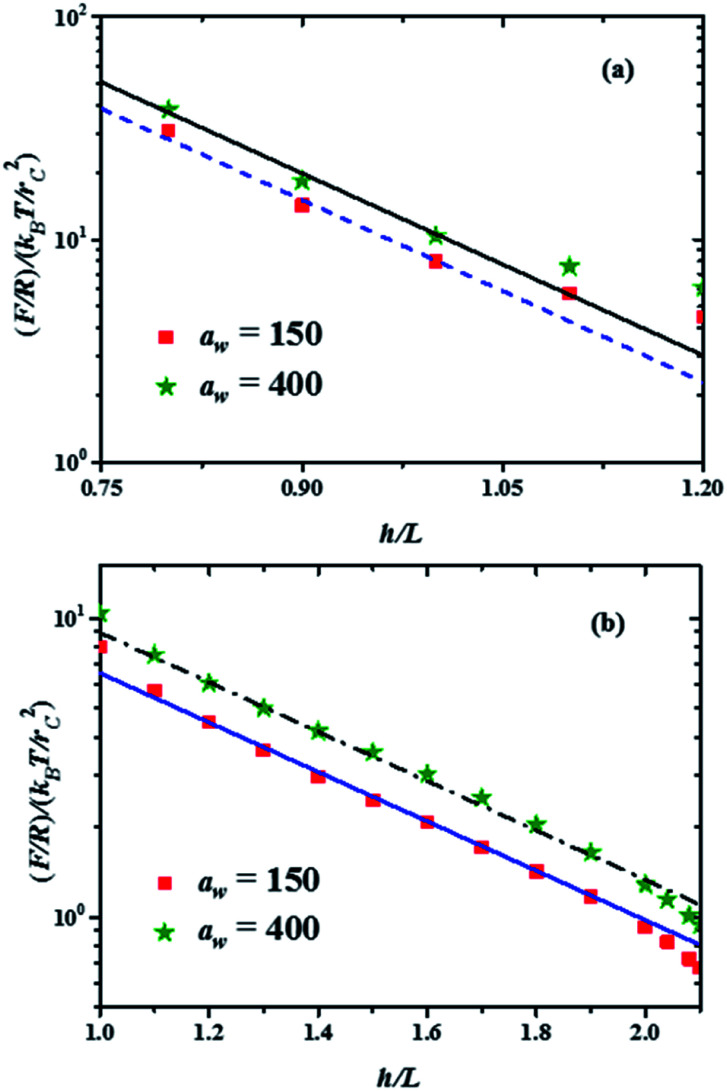
Force profiles in the two compression regimes shown in Fig. 3, for the minimal and maximal stiffness of the grafting surface (*a*_w_, in reduced DPD units). The lines represent the best fits to the minimum and maximum forces in each compression range. The fitting function is *b* exp(−2π*h*/*L*), where *b* is a constant. (a) Force profiles in the strong compression range, with *b* = 5660 for the maximum force profile (continuous black line) and *b* = 4300 for the minimum force profile (dashed blue line). (b) Force profiles in the weak compression regime. The fitting function here is *b* exp(−2π*h*/*L*′), with *L*′ = 3.3*L* and *b* = 60 for the maximum force profile (dash-dotted black line) and *b* = 44 for the minimum force profile (solid blue line).

The properties of the brushes can be modelled with the Alexander–de Gennes (AdG) scaling expression:^34,35^ Π(*h*) = *Γ*^3/2^*k*_B_*T*[(*L*/*h*)^9/4^ − (*h*/*L*)^3/4^] for *h*/*L* < 1, where *Γ* is the brush grafting density. The first term arises from excluded volume repulsion and the second is elastic energy of the chains. For 0.2 < *h*/*L* < 0.9 this expression can be approximated by^32^ Π(*h*) = 100*Γ*^3/2^*k*_B_*T*e^−2π(*h*/*L*)^ and using the Derjaguin approximation one finds *F*/*R* = 100*Γ*^3/2^*k*_B_*TL*e^−2π(*h*/*L*)^. The properties of the surface, such as its stiffness, are not included in these models. The lines in Fig. 4(a) and (b) are best fits to the data using the exponentially decreasing form of the AdG equation *F*/*R* = *b* exp(−2π*h*/*L*), where *b* is a fitting constant.

Good agreement is found with the AdG equation for the brush, regardless the value of *a*_w_. Therefore, properties of the brush, such as *L* remain unchanged by the increasing hardness of the surface on which it is grafted and depend only on the compression. The brush behaves as a nonelastic body and the surface's elasticity propagates through the brush to the compressing surface, giving rise to larger forces for harder surfaces. The ratio between the fits to the maximal and minimal forces in the strong compression regime in Fig. 4(a) yields *r* = *F*(*h*/*L*)_max_/*F*(*h*/*L*)_min_ = 1.32, while for the weak compression regime, Fig. 4(b), *r* = 1.36. Since the force on the brush – covered surface must be proportional to Young's modulus,^36^ it follows that *r* is proportional to the ratio of Young's moduli between the hard and soft surfaces. From Fig. 4 one finds that *r* is approximately the same in both compression ranges, hence the surface's Young modulus is independent of indentation and depends only on the surface stiffness. These conclusions are in agreement with AFM experiments on cell membranes with protrusions that act as a brush.^37^ Guz and co-workers found that the contributions to the force coming from the membrane and the brush could be separated – for a flat AFM tip – if the exponentially decreasing AdG brush model was used and the cell's membrane was described with an effective, indentation – independent Young's modulus.^37^ Using also AFM, Dokukin and collaborators^38^ measured the force on a poly (2-vinylpyridine) substrate with and without a poly (ethylene oxide) brush, changing the stiffness of the substrate with pH. They found that the mechanical response of the substrate and the brush could be accounted for separately, that Young's modulus increased as the substrate became stiffer and that it was independent of the indentation,^38^ in agreement with our results. As for the role of the polydispersity of the brushes, the results show that they only affect the indentation dependence of the force profiles, as depicted in Fig. 3. If the brush is made up of chains of a single polymerization degree, there will be only one compression regime. However, increasing the stiffness of the grafting surface affects the force measured by an AFM tip in a way that does not depend on the polymerization degree, as shown by the experiments^37,38^ and our simulations, see Fig. 4. If the brush is made up of a single chain length, then there is only one compression range and the previous arguments apply. Additional details about the structures of the chains of different polymerization degrees as they are compressed can be found in the ESI.†

To conclude, we performed DPD simulations of a polymer brush made up of chains of three different lengths grafted on a surface whose stiffness was systematically increased. Force-compression curves were obtained when the brush and the solvent were compressed by a flat and featureless surface, representing the tip of an AFM. The force profiles obtained revealed two compression regimes that obeyed well established brush force laws. Increasing the stiffness of the grafting surface led to an increase in the force profile, attributed to an increase in the surface's Young's modulus. The ratio between the force profile for the surface maximum stiffness over that of the minimum stiffness remains approximately constant regardless the compression, which is taken as an indication that Young's modulus is indentation independent, in agreement with AFM measurements on surfaces with brushes.

## Conflicts of interest

There are no conflicts to declare.

## Supplementary Material

RA-010-D0RA01385D-s001
