# Supplementary material for: Mechanical response of a surface of increasing hardness covered with a nonuniform polymer brush: a numerical simulation model
Source: RSC Adv. 2020 Apr 1;10(23):13405–9. doi: 10.1039/d0ra01385d (PMC9051531; doi:10.1039/d0ra01385d)
Supplement: RA-010-D0RA01385D-s001 [file RA-010-D0RA01385D-s001.pdf]

Electronic Supplementary Material (ESI) for RSC Advances.
This journal is © The Royal Society of Chemistry 2020

Electronic Supplementary Information

Mechanical Response of a Surface of Increasing Hardness Covered with a Nonuniform Polymer Brush: A Numerical Simulation Model

J. S. Hernández-Fragoso[a,b], S. J. Alas[a,b,c#] and A. Gama Goicochea[d*]

[a]Posgrado en Ciencias Naturales e Ingeniería, Universidad Autónoma Metropolitana Unidad Cuajimalpa, Ciudad de México 05300, Mexico

[b]Departamento de Ciencias Naturales, Universidad Autónoma Metropolitana Unidad Cuajimalpa, Av. Vasco de Quiroga 4871, Ciudad de México 05300, Mexico

[c]Departamento de Química, Universidad Autónoma Metropolitana Unidad Iztapalapa, Ciudad de México 09340, Mexico

[d]División de Ingeniería Química y Bioquímica, Tecnológico de Estudios Superiores de Ecatepec, Av. Tecnológico s/n, Ecatepec, Estado de México 55210, Mexico

Here we present all the details pertaining the interaction model, the simulation details and additional simulation results.

1. The dissipative particle dynamics (DPD) model

DPD is a particle-based technique [1, 2] which is closely related to standard molecular dynamics [3] in the sense that the equation of motion must be integrated using finite time steps for a number of particles, until equilibrium is achieved for sufficiently long time. What is specific to DPD is the short-range nature of its forces, which are central

* Corresponding authors. Electronic mail: * agama@alumni.stanford.edu, #salas@correo.cuam.uam.mx

and pairwise additive, yielding moment – conserving simulations. Three types of forces make up the DPD model, namely a non-bonding conservative force, a dissipative force, and a random force given by the following expressions, respectively:

$$F_{ij}^{C} = \begin{cases} a_{ij}(1 - r_{ij})\hat{r}_{ij} & r_{ij} \le r_c \\ 0 & r_{ij} > r_c \end{cases} \tag{ESI.1}$$

$$F_{ij}^{D} = -\gamma\omega^{D}(r_{ij})[\hat{r}_{ij} \cdot v_{ij}]\hat{r}_{ij} \tag{ESI.2}$$

$$F_{ij}^{R} = \sigma\omega^{R}(r_{ij})\xi_{ij}\hat{r}_{ij} \tag{ESI.3}$$

In the equations above $\boldsymbol{r_{ij}} = \boldsymbol{r_i} - \boldsymbol{r_j}$, $r_{ij} = |\boldsymbol{r_{ij}}|$, $\hat{r}_{ij} = \boldsymbol{r_{ij}}/r_{ij}$, r_{ij} is the magnitude of the relative position between particles i and j, and a_{ij} is the intensity of the repulsion between a pair of particles. The constant γ is the coefficient of the viscous force, and σ is the amplitude of the random force; $\boldsymbol{v_{ij}} = \boldsymbol{v_i} - \boldsymbol{v_j}$ is the relative velocity between the particles i and j; $\xi_{ij} = \xi_{ji}$ is a random number uniformly distributed between 0 and 1 with Gaussian distribution and unit variance. The weight functions ω^D and ω^R in equations ESI.2 and ESI.3 carry the spatial dependence of those forces and are given by the following expressions:

$$\omega^{D}(r_{ij}) = [\omega^{R}(r_{ij})]^2 = max\left\{\left(1 - \frac{r_{ij}}{r_c}\right)^2, 0\right\} \tag{ESI.4}$$

where r_c is a cut-off distance. At interparticle distances larger than r_c, all forces are equal to zero. This simple distance dependence of the forces allows one to use relatively large integration time steps. The strengths of the dissipative and random forces are related by the dissipation-fluctuation theorem [2], keeping the temperature (T) internally fixed, $k_BT = \frac{\sigma^2}{2\gamma}$; k_B is Boltzmann's constant.

Polymer molecules are modeled as linear chains made of beads connected by freely rotating harmonic springs given by Hooke´s law:

$$F_{ij}^{S} = -k_s(r_{ij} - r_0)\hat{r}_{ij}\ . \tag{ESI.5}$$

The spring constant in all cases modeled in this work is set as $k_s = 100$ and the equilibrium distance at $r_0 = 0.7$ [4]. The surface on which the polymer chains are grafted, and the flat tip of the atomic force microscope (AFM) that compresses the chains are both modeled by a simple, linearly decaying force law [5, 6]:

$$F_w(z_i) = \begin{cases} a_w\left(1 - z_i/z_C\right)\hat{z} & z_i \leq z_c \\ 0 & z_i > z_c \end{cases} \quad . \qquad \text{(ESI.6)}$$

The value of the cutoff distance along the z-axis, z_C is chosen as 1, and a_w is the intensity of the wall – fluid interaction. The component along the z-axis of the position of the i-particle (solvent bead or polymer bead) is labeled z_i and for distances larger than z_C, the wall force is zero. To model the tip of the AFM, the value of a_w in equation (ESI.6) was always kept equal to $a_w = 300\left(k_BT/r_c\right)$. To model an increasingly stiff surface on which the polymer brush is grafted, a_w was increased from $a_w = 150\left(k_BT/r_c\right)$ to $a_w = 400\left(k_BT/r_c\right)$.

2. Simulation Protocol and Details

All simulations were performed using a hybrid DPD – Monte Carlo algorithm [5], so that the simulations could be performed in the grand canonical ensemble, where the chemical potential of the solvent is fixed, as well as the volume of the cell and the temperature. This is necessary to ensure that the system remains in chemical equilibrium when it is compressed, to produce force profiles. Failure to do so (keeping the chemical potential constant) results in erroneous values of the pressure between the surfaces [7, 8]. Full details about the hybrid DPD-Monte Carlo algorithm have been published elsewhere [5].

For the calculation of the force that the tip of the AFM exerts on the surface covered with a brush we use the solvation pressure Π [9]. It is defined as

$$\Pi(h) = P_{zz}(h) - P_B, \qquad \text{(ESI.7)}$$

where $P_{zz}(h)$ is the component of the pressure tensor along the direction of compression (z-axis), i. e., is the pressure exerted on the surfaces that confine the complex DPD fluid made up of polymer chains and solvent monomers. The bulk pressure, P_B, is obtained from the average value of the diagonal components of the pressure tensor when the brush is not compressed. The components of the pressure tensor are calculated using the virial theorem route [3], which provides kinetic and "potential" contributions to the pressure tensor. These components of the pressure tensor were calculated following the model of Irving and Kirkwood [10], given as follows:

$$P_{xx} = \sum_i m_i \vec{v}_i \cdot \vec{v}_i + \sum_i \sum_{j>i} F^C_{ijx} x_{ij} \qquad (ESI.8)$$

where the first sum is the kinetic contribution. The second term is the product of the x – component of the conservative DPD force acting between particles i and j, and the x – component of the r_{ij} vector. The pressure tensor components P_{yy} and P_{zz} are obtained by replacing y and z by x in equation (ESI.8), respectively. Figure ESI.1 shows a schematic representation of the simulation setup.

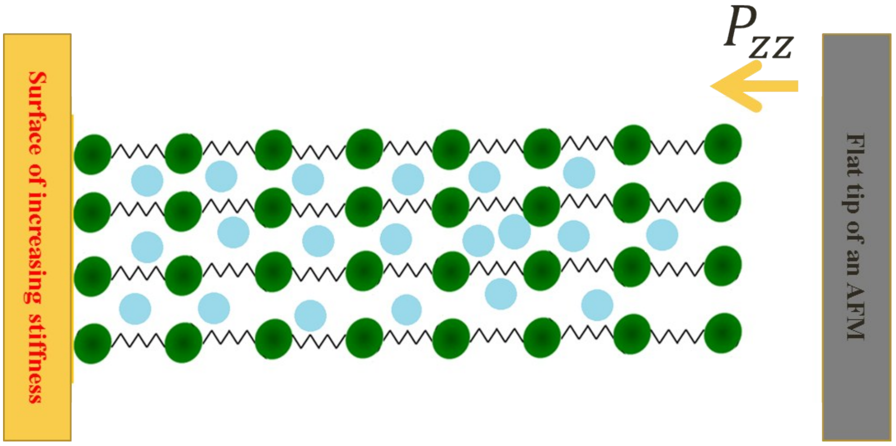

Fig. ESI.1. Illustration of the simulation setup. The surface whose stiffness is increased is shown on the left; the tip of the AFM is on the right. Polymer chains (in green) are grafted to the surface by physical adsorption of the beads at one end of the chains to the surface. For simplicity, all chains shown have the same length. In the simulations we used three values of the length: N_1=5 beads, N_2=30 beads and N_3=42 beads. The solvent beads are represented by blue circles.

We use reduced units throughout this work, where all masses are taken as $m = 1.0$ and the cutoff radius is $r_C = 1.0$. The values chosen for the constants in the random and dissipative forces, σ=3 and γ=4.5, yield $k_BT = 1$ and the time step used to integrate discreetly the equation of motion is set at $\delta t = 0.03$. The parameter of the non-bonding conservative DPD force between two beads, a_{ij} in equation ESI.1, is taken from a previous report [11] equal to $a_{ii} = 78.0(k_BT/r_c)$ for particles of the same type (solvent-solvent, polymer bead-polymer bead). For the interaction between solvent particles and the beads that make up the polymer chains, $a_{ij} = 79.3(k_BT/r_c)$. The interaction between the beads (solvent or polymer bead) and the surface of increasing hardness (a_w in equation ESI.6) was set at $a_w = 140(k_BT/r_c)$. To physically adsorb the chains' end-bead to this surface, a less repulsive wall force was used: $a_w = 60(k_BT/r_c)$; this produces an effective attraction between the end-beads of the chains and their grafting surface. The intensity of the force coming from the tip of the AFM was fixed at $a_w = 300(k_BT/r_c)$ for all types of beads in the system. Following previous works [11, 12] the brush is made up of chains of three polymerization degrees: $N_1 = 5$, $N_2 = 30$ and $N_3 = 42$. For the shortest polymers (N_1) there are 36 chains in the simulation box, with 10 chains of the medium size chains (N_2) and 4 of the longest chains (N_3). When the physical dimensions of the grafting surface are used, these values correspond to grafting densities equal to $\Gamma_1 = 1.76$ nm^{-2}, $\Gamma_2 = 0.49$ nm^{-2} and $\Gamma_3 = 0.20$ nm^{-2}.

The area of the simulation box was fixed in all cases with $L_x/r_c = 7$, $L_y/r_c = 7$, while L_z/r_c was increased from 4.5 up to 15; periodic boundary conditions were used, except in the *z*-direction since this is the direction of the confinement. The chemical potential was fixed at μ=37.7k_BT, which gives a total average numerical density of $<\rho>\sim 3/r_c^3$. The simulation results were obtained from averages after at least 50 blocks of 10^4 Monte

Carlo configurations each were carried out, with the first 25 blocks used to equilibrate the system and the rest were used for the production phase.

3. Additional results

In Fig. ESI.2 we show the concentration profiles of the solvent monomers only in the high compression regime. The global concentration is equal to 3, which means that there are very few solvent particles in the system when the compression is high, yet they do penetrate the brush. Another salient feature is that the hardest the surface becomes (increasing aw), the least solvent beads there are. The surface appears to "push" the beads to the outer end of the brush, close to the AFM tip.

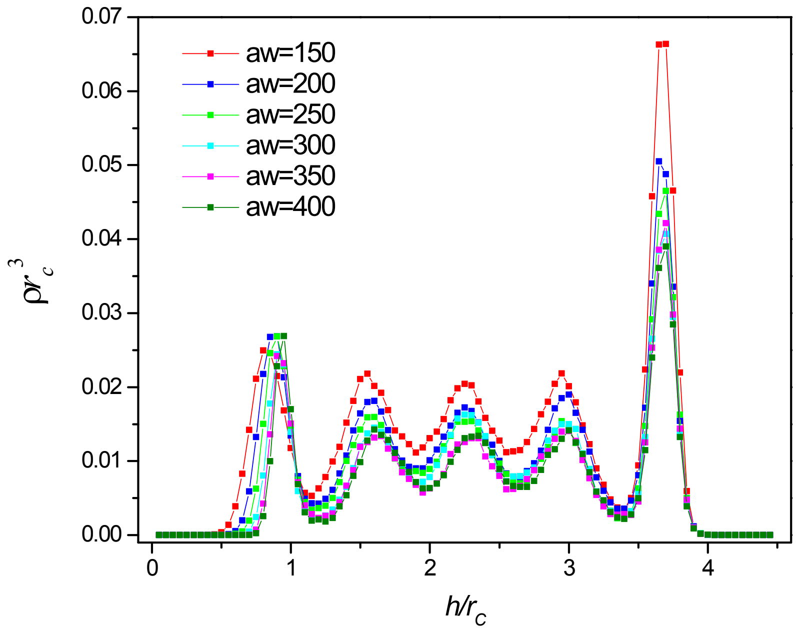

Fig. ESI.2. Concentration profile of the solvent beads only under high compression, when the stiffness parameter of the grafting surface is increased. The grafting surface is on the left *y*-axis while the tip of the AFM is on the right.

In Fig. ESI.3 we show the concentration profiles of the brush beads only, for the weak compression regime. It is of notice that in only one case can we find a structuring of the brush beads near the surface of the AFM tip (*y*-axis on the right), corresponding to

aw=300. The reason for this is quite simple: that value of the grafting surface stiffness (placed on the left y-axis) is equal to the intensity of the force parameter of the AFM tip, therefore the brush beads nearest the tip are locally attracted to such surface.

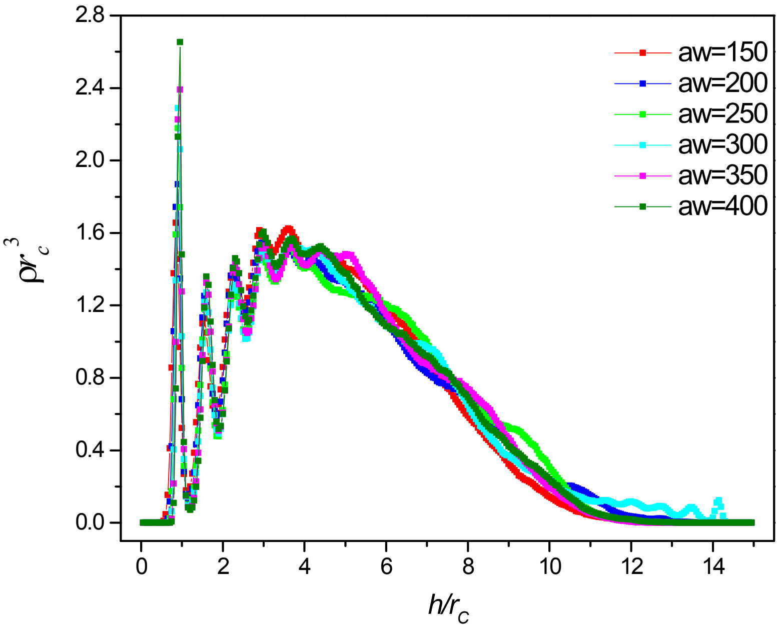

Fig. ESI.3. Concentration profile of the brush beads only as the stiffness parameter of the grafting surface is increased, under weak compression. The grafting surface is on the left y-axis while the tip of the AFM is on the right.

Lastly, we comment on the dependence of the total force exerted on the grafting surface and the brush by the AFM tip when the stiffness of the former is increased. In Fig. ESI.4 one can find the F/R for increasing values of the grafting surface stiffness parameter, a_w, at a fixed distance between both surfaces, $h/L = 1.4$, where L is the average thickness of the nonuniform brush, made up of chains of three different lengths and grafting densities. This distance corresponds to the weak compression regime (see Fig. 4(b) in the main manuscript). The data (blue circles in Fig. ESI.4) follow reasonably well the expression

$$\frac{F}{R} = \beta(a_w)^{\frac{1}{3}} . \qquad \text{(ESI.9)}$$

In the equation above β is a fitting parameter. This figure clearly shows that our simulations pick up separately the contributions to the force arising from the surface and from the brush. That can be ascertained by recalling from the discussion in the main manuscript that the brush mechanical response to compression can be appropriately represented by the Alexander – de Gennes expression [9]:

$$\frac{F}{R} = be^{-2\pi(h/L)}, \qquad \text{(ESI.10)}$$

where b is a constant that depends only on the properties of the brush, $b = 100\Gamma^{3/2}k_BTL$. The fit of equation ESI.10 to the data in Fig. 4 of the manuscript is good, proving that such equation reproduces the force-compression profiles. Yet, there is no contribution from the surface stiffness in equation ESI.10. By fixing the distance between the surfaces ($h/L = 1.4$ in Fig. ESI.4), the properties of the brush are fixed, regardless the value of the stiffness constant, which leads to the conclusion that the trend shown in Fig. ESI.4 is due entirely to the increasing stiffness of the surface.

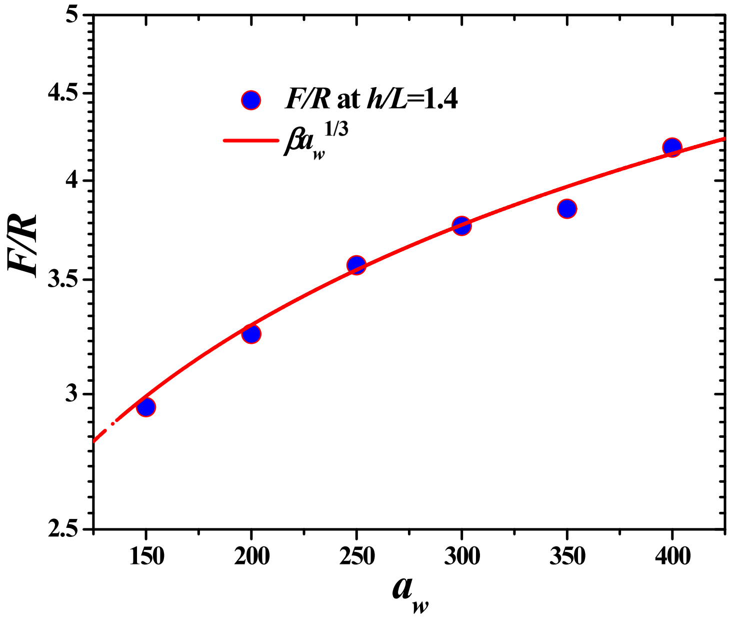

Fig. ESI.4. Dependence of the force on the brush with increasing stiffness of the surface on with the brush is grafted. The red line represents the best fit to the function $\beta a_w^{1/3}$, with $\beta = 0.563$. The scales on the axes are in reduced DPD units.

The results shown in Fig. ESI.4 lead us to postulate that the force applied by the AFM on a surface covered by a nonuniform polymer brush has two uncoupled contributions, namely the brush force, given by equation ESI.10, and the surface force, given by $\beta a_w^{1/3}$. Therefore, latter contribution must be proportional to Young's modulus, $E(a_w) \propto \beta a_w^{1/3}$. This would explain where the ratio $r = F(h/L)_{max}/F(h/L)_{min}$, reported in the main manuscript, comes from. We obtained $r = 1.32$ in the high compression range and $r = 1.36$ in the weak compression range, while $E(a_w = 400)/E(a_w = 150) = (400/150)^{1/3} \cong 1.38$, which is close to value of r.

REFERENCES

[1] Hoogerbrugge, P. J. & Koelman, J. M. V. A. Simulating microscopic hydrodynamic phenomena with dissipative particle dynamics. *Europhys. Lett.* **19**, 155 (1992).

[2] Español, P. & Warren, P. Statistical mechanics of dissipative particle dynamics. *Europhys. Lett.* **30**, 191 (1995).

[3] Allen, M. P. & Tildesley, D. J. *Computer Simulation of Liquids*, Oxford University Press, New York (1989).

[4] Gama Goicochea, A., Romero-Bastida, M. & López-Rendón, R. Dependence of thermodynamic properties of model systems on some dissipative particle dynamics parameters. *Mol. Phys.* **105**, 2375 (2007).

[5] Gama Goicochea, A. Adsorption and disjoining pressure isotherms of confined polymers using dissipative particle dynamics. *Langmuir* **23**, 11656 (2007).

[6] Gama Goicochea, A. & Alarcón, F. Solvation force induced by exact, short range dissipative particle dynamics effective surfaces on a simple fluid and on polymer brushes. *J. Chem. Phys*. **134**, 014703 (2011).

[7] Goujon, F., Malfreyt, P., Tildesley, D. J. Dissipative Particle Dynamics Simulations in the Grand Canonical Ensemble: Applications to Polymer Brushes. *ChemPhysChem*, **5**, 457 (2004).

[8] Balderas Altamirano, M. A., Gama Goicochea, A. Comparison of mesoscopic solvation pressure at constant density and at constant chemical potential. *Polymer* **52**, 3846 (2011).

[9] Israelachvili, J. N. in *Intermolecular and Surfaces Forces* 2nd edn. (Academic Press, 1992).

[10] Irving, J. H., & Kirkwood, J. G. The Statistical Mechanical Theory of Transport Processes. IV. The Equations of Hydrodynamics. *J. Chem. Phys*. **18**, 817 (1950).

[11] Gama Goicochea, A., Alas Guardado, S. J. Computer simulations of the mechanical response of brushes on the surface of cancerous epithelial cells. *Sci. Rep*. **5**, 13218 (2015).

[12] Hernández Velázquez, J. D., Mejía – Rosales, S., Gama Goicochea, A. Nanorheology of pericellular brush models of epithelial normal and cancerous cells using dissipative particle dynamics. *Polymer* **129**, 44 (2017).
